# Integrated elemental analysis supports targeting copper perturbations as a therapeutic strategy in multiple sclerosis

**DOI:** 10.1016/j.neurot.2024.e00432

**Published:** 2024-08-19

**Authors:** James B.W. Hilton, Kai Kysenius, Jeffrey R. Liddell, Stephen W. Mercer, Carsten Rautengarten, Dominic J. Hare, Gojko Buncic, Bence Paul, Simon S. Murray, Catriona A. McLean, Trevor J. Kilpatrick, Joseph S. Beckman, Scott Ayton, Ashley I. Bush, Anthony R. White, Blaine R. Roberts, Paul S. Donnelly, Peter J. Crouch

**Affiliations:** a Department of Anatomy & Physiology, The University of Melbourne, Victoria 3010, Australia; b Florey Institute of Neuroscience and Mental Health, The University of Melbourne, Victoria 3010, Australia; c School of Biosciences, The University of Melbourne, Victoria 3010, Australia; d Atomic Medicine Initiative, University of Technology Sydney, Australia; e School of Chemistry and Bio21 Molecular Science and Biotechnology Institute, The University of Melbourne, Victoria 3010, Australia; f School of Geography, Earth and Atmospheric Sciences, The University of Melbourne, Victoria 3010, Australia; g Elemental Scientific Lasers, LLC, 685 Old Buffalo Trail, Bozeman, MT 59715, United States; h Anatomical Pathology, The Alfred Hospital, Victoria 3004, Australia; i Linus Pauling Institute, Department of Biochemistry and Biophysics, Oregon State University, 97331, United States; j Melbourne Dementia Research Centre, Florey Institute of Neuroscience and Mental Health, The University of Melbourne, Victoria 3010, Australia; k Queensland Institute of Medical Research Berghofer, Herston, Queensland 4006, Australia; l Department of Biochemistry, Emory University, Atlanta, GA 30322, United States

**Keywords:** Multiple sclerosis (MS), Copper, Neuroprotection, CuATSM, Neurodegeneration, Therapeutics

## Abstract

Multiple sclerosis (MS) is a debilitating affliction of the central nervous system (CNS) that involves demyelination of neuronal axons and neurodegeneration resulting in disability that becomes more pronounced in progressive forms of the disease. The involvement of neurodegeneration in MS underscores the need for effective neuroprotective approaches necessitating identification of new therapeutic targets. Herein, we applied an integrated elemental analysis workflow to human MS-affected spinal cord tissue utilising multiple inductively coupled plasma-mass spectrometry methodologies. These analyses revealed shifts in atomic copper as a notable aspect of disease. Complementary gene expression and biochemical analyses demonstrated that changes in copper levels coincided with altered expression of copper handling genes and downstream functionality of cuproenzymes. Copper-related problems observed in the human MS spinal cord were largely reproduced in the experimental autoimmune encephalomyelitis (EAE) mouse model during the acute phase of disease characterised by axonal demyelination, lesion formation, and motor neuron loss. Treatment of EAE mice with the CNS-permeant copper modulating compound Cu^II^(atsm) resulted in recovery of cuproenzyme function, improved myelination and lesion volume, and neuroprotection. These findings support targeting copper perturbations as a therapeutic strategy for MS with Cu^II^(atsm) showing initial promise.

## Introduction

Multiple sclerosis (MS) is a debilitating disease of the central nervous system (CNS) with an unknown aetiology. Pathologically, it is characterised by demyelinated lesions, axonopathy, inflammation and immune involvement in the brain, spinal cord, and optic nerve [1,2]. Most individuals with MS develop a relapsing-remitting form of the disease involving episodes of acute injury and associated disability interspersed with periods of full or partial symptomatic recovery. These patients have recourse to multiple treatment avenues which fall into symptomatic or disease-modifying therapy categories. While the former attempts to ameliorate the burden of symptoms, the latter aims to target immune cells and the inflammatory profile of the disease [1]. Over time, most relapsing-remitting cases transition into a secondary progressive phase of disease where distinct relapse-recovery cycles are replaced by a gradual increase in permanent neurological disability reflecting increasing neurodegeneration. In addition to secondary progressive MS, approximately 10% of all MS patients experience an increase in permanent neurological disability from symptom onset defined as primary progressive MS [2].

Although relapsing-remitting MS presents a significant burden, progressive forms of the disease have the greatest impact on people living with MS and they also suffer from a dearth of available therapeutic options. Once the disease transitions into the secondary progressive stage, the availability of viable therapies and their efficacy diminishes. Ocrelizumab which targets CD20^+^ B cells of the immune system is the only approved treatment with any effectiveness for primary progressive MS. Although partial mitigation of disability was demonstrated for ocrelizumab in patients with primary progressive MS [3,4], addressing neuroprotection and progression remains a significant unmet need in MS drug development [5]. Importantly, as grey matter atrophy associated with neurodegeneration is a principal correlator with disability in all forms of MS [[6], [7], [8]], a neuroprotective approach also has immediate relevance across the spectrum of the disease.

A role for biological metals in MS has been considered for some time, with iron being a particular focus due to its amenability to detection via MRI and histological approaches [[9], [10], [11]]. Besides iron, copper is increasingly recognised as being a potential contributor to disease although earlier studies have been confined to biofluids yielding conflicting findings [12,13]. The relevance of copper in MS is supported by use of the copper-chelating agent cuprizone to model demyelination as well as axonopathy [14,15], an early marker of neurodegeneration. Additionally, a more direct role for copper in MS has recently been proposed whereby aberrant copper trafficking in the white matter could be involved in oligodendrocyte pathology [16]. However, a fuller understanding of how copper changes manifest in MS and whether this represents a prospective therapeutic target in the context of neuroprotection remains to be established.

Here, we applied an integrated elemental analysis approach to quantify the atomic composition of spinal cord tissue from cases of MS. These analyses revealed changes affecting copper as a prominent feature of the disease and coincided with perturbed expression of copper genes and cuproenzyme function. Pharmacological significance of these changes was supported by preliminary assessment of the experimental autoimmune encephalomyelitis (EAE) mouse model of MS in which we show treatment with the CNS-permeant copper modulating compound Cu^II^(atsm) was neuroprotective and restored key pathological events observed in MS.

## Materials and Methods

### Human tissue

Fresh-frozen post-mortem spinal cord tissue collected from human MS cases and controls were obtained from the Victorian Brain Bank (Australia) and the MS Society Tissue Bank (United Kingdom) then stored at -80 ​°C. MS cases used in this study were clinically diagnosed as either primary progressive MS or secondary progressive MS (Additional file 1: Table S1).

### Experimental autoimmune encephalomyelitis (EAE) mouse model

The EAE protocol, utilising female C57BL/6 mice, was based on that of Bittner et al. [17]. Briefly, on day 0 of the study (when the mice were 8 weeks old), mice were anaesthetised via isoflurane inhalation, injected subcutaneously with 200 ​μL MOG_35-55_ peptide (Pepceuticals) prepared at 1 ​mg ​mL^-^^1^ in complete Freund's Adjuvant (Sigma), then intraperitoneally injected with 400 ​ng pertussis toxin (Enzo Life Sciences). A second dose of 400 ​ng pertussis toxin was injected two days later. Control mice were anaesthetised, injected with complete Freund's Adjuvant, and received two injections of pertussis toxin as per EAE mice, except the complete Freund's Adjuvant did not contain any MOG_35-55_ peptide.

Prior to inducing the EAE phenotype, mice were randomly allocated into one of three treatment groups: control, EAE, and EAE plus Cu^II^(atsm). Mice in the EAE plus Cu^II^(atsm) group were treated with Cu^II^(atsm) by gavage at a dose of 30 ​mg ​kg^-^^1^ body weight, once daily. Cu^II^(atsm) was synthesised as described previously [18,19] and prior to gavage a fine suspension at 7.5 mg mL^-1^ was prepared in standard suspension vehicle (SSV; 0.9% w/v NaCl, 0.5% w/v Na-carboxymethylcellulose, 0.5% v/v benzyl alcohol, 0.4% v/v Tween 80) each day via sonication. Mice in the control and EAE groups were sham gavaged with an equivalent volume of SSV that did not contain Cu^II^(atsm). Gavage of all mice commenced 5 days after the MOG_35-55_ peptide injection. Signs of neurological symptoms were assessed using the previously reported clinical scoring system [17], with researchers monitoring symptom score being blinded to the treatments administered by a separate researcher.

On day 15 of the study period mice were deeply anaesthetised by intraperitoneal injection of saline solution supplemented with ketamine and xylazine (120 and 16 ​mg ​kg^-^^1^ body weight respectively) then transcardially perfused with PBS supplemented with phosphatase inhibitors (Phosphatase Inhibitor Cocktail 2; Sigma), protease inhibitors (Complete EDTA-free Tablets (Roche), and heparin (20 U ​mL^-^^1^). After perfusion, mice were dissected to remove spinal cords with collected tissue either snap frozen and stored at -80 ​°C or fixed for subsequent histological and electron microscope examination as described below.

### Tissue processing for biochemical analyses

Fresh-frozen spinal cord tissue samples (human tissue and EAE model) were homogenised using polypropylene pestles in tris-buffered saline (TBS) supplemented with 0.5% (v/v) phosphatase inhibitor cocktail 2 (Sigma), 2% (w/v) Complete EDTA-free protease inhibitor (Roche), and 5% (v/v) DNase. Homogenates were then separated into TBS-soluble fractions and TBS-insoluble pellets by centrifugation (21,000 RCF, 4 ​°C) for 30 ​min. Insoluble pellets were resuspended in the same TBS-based homogenisation buffer and re-homogenised using polypropylene pestles to produce TBS-insoluble suspensions. Protein content of TBS-soluble and -insoluble fractions was determined using the BCA Assay (Thermo Fisher Scientific) then all samples normalised to a consistent protein concentration using the TBS homogenising buffer described above for TBS-soluble and TBS-insoluble fractions. As previously shown for mouse spinal cord samples [20], this protocol for separating TBS-soluble and TBS-insoluble material enriches for cytosolic and small organelle proteins in the TBS-soluble fraction and membrane, nuclear, and cytoskeletal proteins in the TBS-insoluble fraction (Additional file 1: Fig. S1).

### Inductively coupled plasma-mass spectrometry (ICP-MS)

Weighed spinal cord tissue samples were digested in 65% (v/v) nitric acid prior to addition of an equivalent volume of 30% (v/v) H_2_O_2_ then diluting in 1% (v/v) nitric acid. Quantitation of elemental constituents utilised an Agilent 7700 Series ICP-MS under routine multi-element ICP-MS conditions and a helium reaction cell gas. The instrument was calibrated using certified multi-element ICP-MS calibration solutions and yttrium (^89^Y) was used as an internal control. High abundance elements measured in analysed samples (^23^Na, ^24^Mg, and ^95^Mo) were used as a multi-element normalisation factor. Total protein content of the same samples prepared in parallel enabled expression of results as μg element g^-^^1^ protein.

### Laser ablation-inductively coupled plasma-mass spectrometry (LA-ICP-MS)

To measure levels of copper partitioned into different sample fractions, 0.5 ​μL aliquots of TBS-soluble and insoluble fractions from spinal cord tissue were loaded onto microscope slides then allowed to air-dry [21]. Equivalent volumes of TBS homogenising buffer were loaded onto the slides as a matrix control. Standards consisting of known copper concentrations made up in TBS homogenising buffer or TBS were also loaded onto slides for subsequent analyses. Quantitation of copper levels across different anatomical regions was performed using fresh-frozen spinal cord tissue. A cryostat was used to cut 30 ​μm thick transverse tissue sections which were then mounted onto microscope slides.

All sample slides were air-dried overnight then imaged and analysed using LA-ICP-MS as described previously [22]. In short, slides were placed in a 10 ​× ​10 ​cm ablation cell together with matrix-matched elemental standards. Sample fractions were then ablated using a 40 ​μm square laser spot size at a scanning speed of 160 ​μm ​s^-^^1^ using the NWR213 ablation system (Kennelec Scientific). The resultant ablated material was transferred into the 8800 QQQ-ICP-MS (Agilent) using an argon gas flow at 1.2 ​L ​min^-^^1^.

Data were analysed using the Iolite analysis software package operating under the Igor Pro suite, with the carbon (^13^C) channel used for signal normalisation. Images used for quantitation were constructed for ^63^Cu. The resultant values were then corrected to total copper levels based on the combined abundance of physiologically relevant stable isotopes (^63^Cu and ^65^Cu). Total protein content of the same samples prepared in parallel enabled calculation of results in TBS-soluble and -insoluble fractions as μg copper g^-^^1^ protein. Data for *in situ* quantitation were calculated as μg copper g^-^^1^ tissue.

### SDS-PAGE and western blotting

For detection of ceruloplasmin, hephaestin, dopamine β-hydroxylase (DβH), lysyl oxidase (LOX), lysyl oxidase like-3 (LOXL3), and 2′,3′-cyclic-nucleotide 3′-phosphodiesterase (CNPase), TBS-insoluble fractions were either used directly or supplemented with 1% (v/v) triton X-100 and mixed before centrifugation (18,000 RCF, 4 ​°C) for 5 ​min to produce triton X-100 soluble fractions. For detection of myelin oligodendrocyte glycoprotein (MOG), histone H3, β-tubulin, CNPase, β-actin, citrate synthase, lysosomal associated membrane protein 1 (LAMP1), and GAPDH, TBS-soluble and TBS-insoluble fractions were used. TBS-soluble, TBS-insoluble and triton X-100 soluble fractions were added to reducing and denaturing sample buffer containing 62.2 ​mM Tris, 5% (v/v) glycerol, 2% (w/v) SDS, and 0.0025% (w/v) bromophenol blue, then heated at 95 ​°C for 5 ​min. Samples were then loaded onto 4–12% NuPAGE Novex Bis-Tris Midi gels (Life Technologies) and resolved by electrophoresis at 200 ​V for 40 ​min in MES SDS running buffer (Life Technologies). Resolved proteins were transferred onto PVDF membranes using iBlot gel transfer stacks (Life Technologies) as per manufacturer's instructions. Membranes were then incubated for 1 ​h in blocking buffer consisting of phosphate buffered saline (PBS) supplemented with 0.05% (v/v) Tween 20 (Chemsupply) and 4% (w/v) skim milk powder before incubation with primary antibodies in blocking buffer overnight at 4 ​°C.

Membranes of resolved proteins were probed with primary antibodies raised to detect ceruloplasmin (DAKO Q0121; 1:1000), hephaestin (Santa Cruz sc-365365; 1:200), DβH (Abcam ab96615; 1:1000), LOX (Abcam ab1743316; 1:1000), LOXL3 (Aviva Systems Biology ARP60280-P050; 1:500), CNPase (Abcam ab6319, 1:500), MOG (Abcam ab109746, 1:1000), histone-H3 (Cell Signaling Technology #9715, 1:1000), β-tubulin (Cell Signaling Technology #2146; 1:3000), β-actin (Cell Signaling Technology #3700; 1:2000), citrate synthase (Abcam ab96600, 1:1000), LAMP1 (Sigma SAB3500285, 1:1000), or GAPDH (Cell Signaling Technology #2118; 1:5000). Horseradish peroxidase conjugated secondary antibodies for anti-rabbit IgG (Cell Signaling Technology #7074; 1:5000) or anti-mouse IgG (Cell Signaling Technology #7076; 1:5000) were made up in blocking buffer and used to incubate relevant membranes. Membranes were incubated in Enhanced Chemiluminescence (ECL Advance; GE Healthcare) to visualise immunoreactive protein bands, and images were taken using the FujiFilm LAS-3000 imager. Densitometric quantitation of protein bands was performed on ImageJ software using TIFF images.

### Ferroxidase activity assay

Ferroxidase activity was measured in protein-normalised triton X-100 soluble fractions using the previously described ferroxidase assay [23,24]. Prior to each assay, fresh aliquots of 250 ​μM human apotransferrin (Sigma) and 1 ​mM FeSO_4_ were prepared with N_2_ gas-purged dH_2_O to mitigate ferroxidase-independent oxidation of iron. Reaction mixtures were loaded into a 96-well plate and consisted of triton X-100 soluble sample, 25 ​mM HEPES, 75 ​mM NaCl, and 50 ​μM human apotransferrin (pH 7.2), then the reaction was initiated by adding FeSO_4_ to a final concentration of 100 ​μM.

Ferroxidase activity was determined via plate reader by calculating the rate of change of absorbance at 460 ​nm through the reaction linear phase. Activity levels were presented as amount of holo-diferric transferrin produced per minute per mg of sample protein (extinction coefficient, 4.56 ​mM^-^^1^ ​cm^-^^1^) using Beer's law. Wells containing equivalent volumes of TBS supplemented with 1% (v/v) triton X-100 in the absence of sample protein were used to control for non-specific activity.

### DβH activity assay

DβH activity in human samples was measured from triton X-100 soluble fractions using liquid chromatography tandem mass spectrometry (LC-MS/MS) to monitor enzymatic production of the DβH product norepinephrine. Triton X-100 soluble samples were added to individual microfuge tubes then combined with a reaction mixture containing 200 ​mM sodium acetate (pH 5.0), 30 ​mM N-ethylmaleimide, 5 ​μM CuSO_4_, 50 ​μL ​mL^-^^1^ catalase (Sigma), 10 ​mM sodium fumarate, and 10 ​mM ascorbate. Following pre-incubation at 37 ​°C for 5 ​min, reactions were initiated by adding 10 ​mM dopamine and then incubated at 37 ​°C for 45 ​min.

A known concentration of epinephrine was added to each sample tube as an internal standard, followed by 2 ​mL 100 ​mM ammonium dihydrogen phosphate (pH 10) supplemented with 2% (v/v) stabiliser (0.5 ​M EDTA, 317 ​mg ​mL^-^^1^ sodium metabisulfite). Each sample was then subject to solid phase extraction (SPE) using Bond Elut phenylboronic acid 100 ​mg, 3 ​mL cartridges (Agilent). Cartridges were equilibrated with 1 ​mL acetonitrile followed by 1 ​mL 5% (v/v) formic acid made up in methanol then 100 ​mM ammonium dihydrogen phosphate (pH 10.0). After sample addition, the matrix was washed sequentially with 2 ​mL 1% (v/v) ammonium hydroxide in 95% (v/v) methanol, 2 ​mL 1% (v/v) ammonium hydroxide in 95% (v/v) acetonitrile, then 1% (v/v) ammonium hydroxide in 30% (v/v) acetonitrile. Once the matrix was dried under vacuum, analytes were eluted using 3 x 500 ​μL aliquots of 5% (v/v) formic acid in methanol and then evaporated in a vacuum concentrator before being reconstituted in 0.3% (v/v) formic acid made up in dH_2_O.

LC-MS/MS analyses were performed using a 1100 series HPLC system (Agilent), and a 4000 QTRAP LC-MS/MS system (Sciex) equipped with a TurboIonSpray ion source. The system was run in Micro mode using a mix rate of 400 ​μL ​min^-^^1^, with the column compartment set to 50 ​°C and samples kept at 20 ​°C. Catecholamine analytes were separated using a Hypercarb column (150 ​mm ​× ​1 ​mm, 5 ​μm particle size, Thermo Fisher Scientific) at a flow rate of 50 ​μL ​min^-^^1^. Initial run conditions used 99% buffer A (0.3% (v/v) formic acid in dH_2_O) and 1% buffer B (100% acetonitrile) for 1 ​min followed by a gradient to 25% buffer B within 20 ​min, then 80% buffer B within 2 ​min. Conditions were then held at 80% buffer B for 2 ​min before a return to 1% buffer B within 2 ​min and holding at 1% buffer B for 6 ​min.

The QTRAP was set to positive ion mode using the multiple reaction monitoring (MRM) scan type, and conditions were spray voltage set to 4200 ​V, source temperature set to 425 ​°C, collision gas set to high, with source gas 1 and source gas 2 set to 20. A time of 100 ​ms was applied to each transition resulting in a duty cycle of 1.0501 ​s, with Q1 and Q3 resolutions set to Unit. Data were collected using the Analyst 1.5.1 Build 5218 (Sciex) operating in MRM mode. Compound-dependent MS parameters are specified in Additional file 1: Table S2. Catecholamine analytes were quantified using the MultiQuant 2.1 (build 2.1.1296.02.1) software package (Sciex) through integration of signal peaks for norepinephrine, dopamine, and epinephrine. Activity levels were calculated with respect to norepinephrine levels following reactions and presented as amount of norepinephrine produced per minute of reaction incubation per mg of sample protein.

DβH activity in EAE model spinal cord samples was determined as per human samples with the following conditions for sample preparation and analysis. SPE was performed using a Bond Elut phenylboronic acid 96 well plate with 100 ​mg bed mass, 2 ​mL well volume (Agilent). SPE sample preparation was facilitated using the Waters Positive Pressure-96 Processor on a low pressure setting. Wells were equilibrated with 750 ​μL acetonitrile followed by 750 ​μL 5% (v/v) formic acid in methanol and then 1 ​mL 100 ​mM ammonium dihydrogen phosphate (pH 10.0). After addition of samples, the wells were washed with 1.2 ​mL 1% (v/v) ammonium hydroxide in 95% (v/v) methanol, followed by 1.2 ​mL 1% (v/v) ammonium hydroxide in 95% (v/v) acetonitrile, then 1% (v/v) ammonium hydroxide in 30% (v/v) acetonitrile. After drying the matrix under vacuum, sample analytes were eluted with 1.5 ​mL 5% (v/v) formic acid in 75% (v/v) acetonitrile to facilitate freeze drying. Samples were then reconstituted in 0.05% (v/v) trifluoroacetic acid (TFA) containing 10 ​μM ascorbate to prevent oxidation.

LC/MS analyses were performed using a 1260 Infinity II HPLC system (Agilent) and a 6120 single quadrupole LC/MS system (Agilent) with an ElectroSpray Ionisation source. The HPLC system was set for an eject speed of 400 ​μL ​min^-^^1^ with column oven set to 50 ​°C. Analytes were separated using a ZORBAX Eclipse Plus Phenyl-Hexyl column (100 ​mm ​× ​4.6 ​mm, 5 ​μm particle size; Agilent) fitted with a ZORBAX Eclipse Plus Phenyl-Hexyl guard column (12.5 ​mm ​× ​4.6 ​mm, 5 ​μm particle size; Agilent) at a flow rate of 0.4 ​mL ​min^-^^1^. Initial run conditions used 99% mobile phase A (0.3% (v/v) formic acid in HPLC grade water) and 1% mobile phase B (0.05% (v/v) TFA in acetonitrile) for 1 ​min, followed by a gradient to 25% mobile phase B within 20 ​min, then 80% mobile phase B within 2 ​min. These conditions were held for 2 ​min before returning to 1% mobile phase B within 2 ​min and holding at 1% mobile phase B for 6 ​min.

The LC/MS was set to positive ion mode with a mass range of 100–1000 *m*/*z* and gas temperature set to 250 ​°C, drying gas flow rate at 12 ​L ​min^-^^1^, nebuliser pressure at 1811 ​Torr, and capillary voltage at 4000 ​V. Data were collected using the ChemStation software package (Agilent) with chromatograms confirmed as analyte of interest by corresponding product ion mass spectra with individual samples quantified for levels of norepinephrine, epinephrine, and dopamine by integration of signal peaks. Human tissue samples quantified by LC-MS/MS were also analysed and used for standardisation where for all analyses norepinephrine levels were normalised to the epinephrine internal standard.

### LOX activity assay

Activity for LOX and LOXL3 proteins was measured based on a previously described fluorometric assay detecting the generation of resorufin from the substrate Amplex UltraRed (Thermo Fisher Scientific) in response to LOX/LOXL-mediated H_2_O_2_ production [25]. Suspensions of TBS-insoluble material from human and EAE model spinal cord samples were centrifuged to isolate TBS-insoluble pellets. Pelleted material was resuspended in a 50 ​mM borate buffer containing 6 ​M urea supplemented with 0.5% (v/v) phosphatase inhibitor cocktail 2 (Sigma), 2% (w/v) Complete EDTA-free protease inhibitor (Roche) made up in dH_2_O (pH 8.2). Samples were homogenised thoroughly and left at 4 ​°C overnight on a rocker to maximise LOX and LOXL3 extraction before centrifugation to retrieve a urea-soluble fraction.

Samples were aliquoted into tubes containing a reaction mixture with final concentrations of 50 ​mM borate, 1.2 ​M urea, 10 ​mM 1,5-diaminopentane dihydrochloride, 1 ​U mL^-1^ horseradish peroxidase, and 20 ​μM Amplex UltraRed (pH 8.2). Parallel sample tubes containing 500 ​μM BAPN as an inhibitor were used to detect activity attributable directly to LOX/LOXL proteins. All samples were vortexed and placed on a heat block away from light at 37 ​°C for 30 ​min then placed on ice to stop the reaction before loading onto 96-well plates containing wells with known concentrations of H_2_O_2_ used as standards. Wells containing reaction mixture without sample were also used as a negative control. End-point fluorescence (Ex 563 ​nm/Em 587 ​nm) was used to calculate activity with reference to H_2_O_2_ standards.

### RNA extraction and transcript analyses

Transverse sections of human spinal cord were used to visually separate grey and white matter using a scalpel and performed on dry ice. Mouse spinal cord and spleen samples were processed whole. All reagents were from Thermo Fisher Scientific unless otherwise indicated and used in accordance with manufacturer's instructions. RNA was isolated from tissue samples using TRI Reagent (Sigma). Contaminating DNA was degraded by treatment of isolated RNA with DNase (Turbo DNA-free Kit). RNA quantity was determined by nanodrop or Qubit RNA HS Assay Kit. cDNA was synthesised using High-Capacity cDNA Reverse Transcription Kit.

cDNA (25 ​ng) was pre-amplified for all genes assessed using Taqman PreAmp Master Mix and pooled Taqman Gene Expression Assays (Additional file 1: Table S3). Pre-amplified cDNA was then diluted 20-fold for subsequent analyses. Spleen cDNA was run directly for all genes without pre-amplification using Taqman Gene Expression Assay (Additional file 1: Table S4). All samples were run in triplicate per gene. Quantitative RT-PCR was performed using Taqman Gene Expression Assays and Taqman Fast Advanced Mastermix on a QuantStudio 6 Flex system (Thermo Fisher Scientific). Relative gene expression was determined via the ΔΔ-ct method normalised to *GAPDH* (human tissue) or *Gapdh* (mouse tissue) expression.

### Microscopy

Fresh lumbar spinal cord samples collected from mice were post-fixed in PBS containing 4% (v/v) paraformaldehyde (PFA) for at least 24 ​h. PFA fixed spinal cord samples were then embedded in paraffin and cut to produce 7 ​μm sections for Luxol fast blue staining of myelin. In brief, sections were deparaffinised in xylene and hydrated in ethanol sequentially before overnight incubation at 60 ​°C in Luxol fast blue solution (0.1% (w/v) Luxol fast blue, 0.05% (v/v) glacial acetic acid, 95% (v/v) ethanol). Slides were then washed briefly in ethanol and differentiated in lithium carbonate solution containing 0.05% (w/v) lithium carbonate before a wash in deionised water and counterstaining with 0.1% (w/v) cresyl violet solution. Sequential dehydration in ethanol and clearing in xylene was done before mounting slides and imaging using CaseViewer (3DHISTECH, version 2.7).

Additional mouse spinal cord samples were cut transversely into 1 ​mm slices and fixed in glutaraldehyde buffer (2.5% (v/v) glutaraldehyde, 2% (w/v) PFA, 0.1 ​M cacodylate buffer) overnight at 4 ​°C. Tissues were then rinsed 3 times with 0.1 ​M cacodylate buffer, incubated in 1% (w/v) osmium tetroxide and 1.5% (w/v) potassium ferrocyanide in distilled water for 2 ​h, then rinsed with distilled water and stored overnight at 4 ​°C. Tissues were next dehydrated through a series of ethanol and acetone, followed by infiltration and embedding in Spurr's resin and polymerised overnight at 70 ​°C.

To quantify ventral motor neurons and white matter lesion volumes, semi-thin sections (0.5 ​μm thick) were stained with toluidine blue and digital images of light microscopy collected using a Mirax Digital Slide Scanner. All images were examined using CaseViewer (3DHISTECH, version 2.7) and analysis of exported images performed using ImageJ (http://rsbweb.nih.gov/ij/). Motor neurons were determined by measuring soma perimeters then calculating diameter based on assumed circularity; only motor neurons with a calculated diameter >13 ​μm were included in final analyses. An average of 121 neurons from 5-6 sections per mouse were counted. White matter lesion volume was calculated as the total lesion area expressed as a percentage of the total white matter area.

To examine axonal diameter and myelin sheath thickness, ultrathin sections (90 ​nm) were stained with uranyl acetate and lead citrate and examined by transmission electron microscopy (JEOL1101, Inc., USA). Electron microscope images were taken with a Megaview III FW (Olympus Soft Imaging Solutions, Münster, Germany) camera equipped to the microscope. Images were quantified using ImageJ (http://rsbweb.nih.gov/ij/) with myelin thickness and axonal diameter determined by measuring total outer diameter (d_o_; inclusive of myelin sheath) and inner diameter (d_i_; exclusive of myelin sheath), with measurements performed on two perpendicular planes per axon and the average per axon used in final analyses. An average of 115 axons was counted per animal.

### Statistical analyses

All statistical analyses were performed using GraphPad Prism Version 9. Prior to performing statistical analyses to compare mean differences between groups, data were assessed for outliers using ROUT [26] and identified outliers excluded from further analysis. Data were then tested for normal distribution and statistical significance of observed differences between group means was assessed using the two-tailed Student's *t*-test, Mann-Whitney test, or ordinary one-way ANOVA with Šídák's multiple comparisons test. Statistical significance was determined as P ​< ​0.05.

## Results

**Fig. 1:**
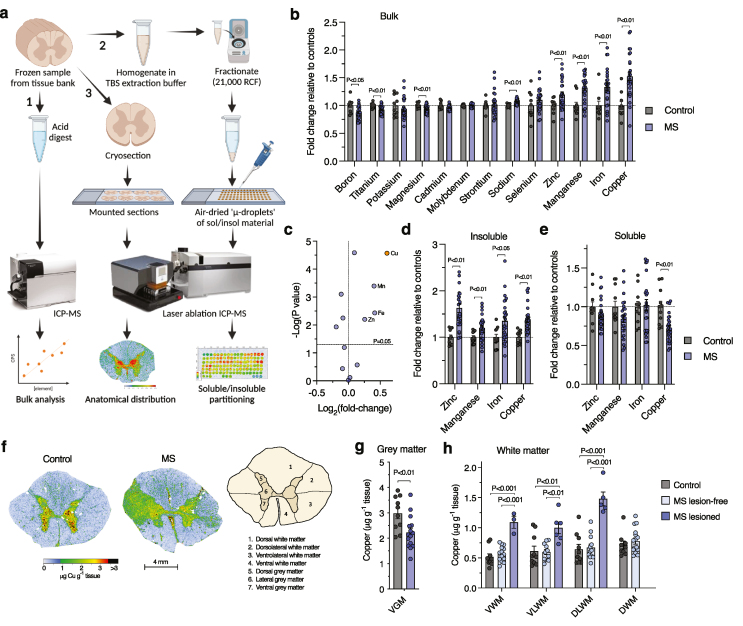
**Integrated elemental analysis workflow applied to the human MS-affected spinal cord**. (a) Illustration of workflow for quantifying: 1. Total tissue element content (bulk analysis); 2. Elemental partitioning in fractions; 3. Anatomical distribution. Bulk analyses utilised direct injection via inductively coupled plasma-mass spectrometry (ICP-MS). Elemental partitioning and anatomical distribution analyses utilised samples on microscope slides assessed via laser ablation-ICP-MS (LA-ICP-MS). (b) Bulk tissue content of endogenous elements in the spinal cord of MS cases expressed relative to controls. (c) Volcano plot of data from (b) with elements having a fold change ≥1.2 labelled and copper (>1.5-fold change) highlighted. (d, e) Fraction partitioning of endogenous zinc, manganese, iron, and copper into TBS-soluble and -insoluble fractions for MS cases expressed relative to controls. (f) Representative LA-ICP-MS images for *in situ* quantitation of copper performed on transverse sections of fresh-frozen spinal cord. Illustration highlights anatomical regions of interest for quantitation. (g, h) Copper levels in the spinal cord ventral grey matter and white matter regions with or without inferred lesions (VWM: ventral white matter; VLWM: ventrolateral white matter; DLWM: dorsolateral white matter; DWM: dorsal white matter). Circles in bar graphs represent individual MS and control cases. Bar graphs presented as mean values ​± ​SEM with labelled P values indicating statistically significant differences.

Changes in the elemental composition of biological material provide unique insights to subjects ranging from mobility of extinct megafauna [27] to objective delineation of tumours from healthy tissue [28]. In this study, we applied an integrated elemental analysis methodology to human spinal cord tissue from MS cases (Fig. 1a; Additional file 1: Table S1). For initial elemental analyses, frozen spinal cord samples were digested in nitric acid and total tissue content of 13 endogenous elements quantified using inductively coupled plasma-mass spectrometry (ICP-MS). Eight of the elements measured were changed in MS cases relative to controls (Fig. 1b; Additional file 1: Fig. S2a) where four displayed a fold-change ≥1.2 (zinc, manganese, iron and copper) with copper showing the greatest change at 1.52-fold over controls (Fig. 1c).

To provide greater insight into potential alterations in elemental distribution, frozen spinal cord samples from the same cases were homogenised in a TBS-based extraction buffer. Partitioning of zinc, manganese, iron, and copper into TBS-soluble and TBS-insoluble fractions was then determined using a laser ablation ICP-MS (LA-ICP-MS) microdroplet approach for quantifying endogenous elements in low volumes of biological samples [21]. These analyses revealed that accumulations in bulk metals content were driven by increased presence in TBS-insoluble material (Fig. 1d; Additional file 1: Fig. S2b). Notably, microdroplet analyses showed that the accumulation of copper in the TBS-insoluble fractions was accompanied by a 28% decrease in the TBS-soluble fraction (Fig. 1e; Additional file 1: Fig. S2c). In addition, no relationship was observed between copper levels and case age (Additional file 1: Fig. S3) demonstrating that these copper changes are related to disease. None of the other elements that displayed a fold-change ≥1.2 in the TBS-insoluble fraction were changed in the TBS-soluble fraction. Thus, copper was the only element to exhibit changes in MS cases across both fractions.

To further examine these copper changes, we anatomically mapped human spinal cord copper levels by applying LA-ICP-MS to cryo-sectioned samples. These analyses uncovered that copper is naturally enriched within the grey matter regions of the human spinal cord and that MS cases are characterised by a diminution of copper in the ventral grey matter but not other grey matter regions (Fig. 1f and g; Additional file 1: Fig. 4a and b). Grey matter atrophy related to neurodegeneration is strongly correlated with disability in MS [[6], [7], [8]] and the ventral grey matter is the anatomical site of lower motor neuron loss in MS [29]. In contrast to the ventral grey matter, MS spinal cord white matter regions displayed a notable elevation in copper. Although overall copper levels in these regions did not significantly differ from controls (Additional file 1: Fig. S4c), a number of MS cases showed localised and distinctly demarcated areas of copper accumulation (Fig. 1f–h). The presence of lesions was inferred from these areas of elevated copper based on analogous findings of iron accumulation detected in MS lesions using LA-ICP-MS [30] and supported by histological evidence for upregulation of the copper transporters CTR1, ATP7A, and ATP7B in both active and chronic inactive MS lesions [16]. Collectively, these elemental analyses show perturbations to endogenous copper as a conspicuous feature of MS.

**Fig. 2:**
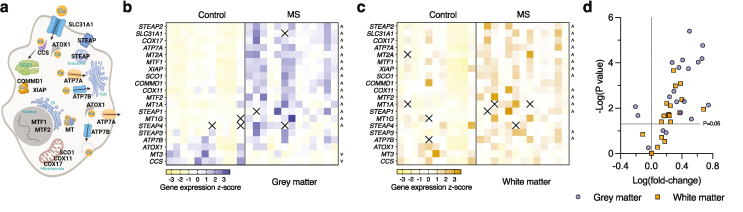
**Altered expression of copper handling genes in the human MS-affected spinal cord**. (a) Cellular illustration of measured gene products involved in copper handling. (b, c) Z-score heatmaps showing expression of genes encoding copper transporters and chaperones in spinal cord grey and white matter measured by quantitative RT-PCR. Squares in transcript heatmaps represent z-scores for individual control and MS cases. Up and down arrowheads identify genes with significantly increased or decreased expression in MS cases, respectively. Crosses in the heatmaps represent excluded values. (d) Volcano plot of data from (b, c) highlighting genes that are significantly up- or down-regulated using a P ​< ​0.05 threshold labelled as a dotted line.

Concordant with disrupted copper levels producing a biological impact, 19 of 20 copper handling genes analysed in human spinal cord tissue displayed altered expression in MS cases compared to controls (Fig. 2; Additional file 1: Figs. S5a and b). Most of these genes involved increased expression in both the MS white and grey matter, with only *MT3* and *CCS* displaying decreased expression specifically in the grey matter. While MS white and grey matter transcript profiles displayed evident similarities despite contrasting levels of copper, 8 genes showed more pronounced expression in the grey matter compared to white matter (Additional file 1: Fig. S5c).

**Fig. 3:**
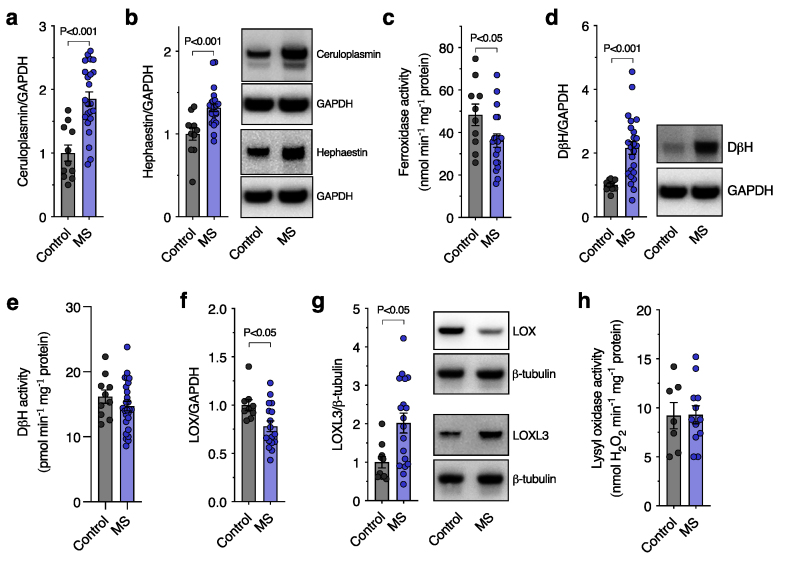
**Altered functionality of cuproenzymes in the human MS-affected spinal cord**. (a, b) Protein levels of the ferroxidases ceruloplasmin and hephaestin determined by western blot and expressed relative to control cases. (c) Ferroxidase activity in control and MS spinal cord samples expressed as nmol holo-transferrin produced min^-^^1^ mg^-^^1^ sample protein. (d) DβH protein levels determined by western blot and expressed relative to control cases. (e) Activity of DβH in control and MS cases expressed as pmol norepinephrine produced min^-^^1^ mg^-^^1^ sample protein. (f, g) Protein levels of LOX and LOXL3 determined by western blot and expressed relative to control cases. (h) Total lysyl oxidase activity in control and MS cases expressed as nmol H_2_O_2_ produced min^-^^1^ mg^-^^1^ sample protein. Circles in bar graphs represent individual MS and control cases. Bar graphs presented as mean values ​± ​SEM with labelled P values indicating statistically significant differences.

These gene expression changes indicated perturbations to spinal cord copper were associated with changes in the molecular machinery that governs the distribution of cellular copper. To understand the downstream functional impact of these alterations we next measured protein levels and activity of copper-dependent cuproenzymes. Although the multi-copper ferroxidases ceruloplasmin and hephaestin involved in cellular iron efflux [31] were increased in the MS spinal cord (Fig. 3a and b), overall ferroxidase activity was decreased (Fig. 3c), indicating a mismatch between levels and functional output. Analogous changes were observed for the neurotransmitter synthesis cuproenzyme dopamine-β-hydroxylase (DβH) with protein levels increased without a commensurate increase in copper-dependent activity (Fig. 3d and e). Assessment of the lysyl oxidase (LOX) family, involved in extracellular matrix remodelling in response to tissue damage [32], revealed decreased protein levels for LOX and increased levels for LOXL3 (Fig. 3f and g). These changes were found to correspond to unchanged overall LOX activity (Fig. 3h) which accounts for both LOX and LOXL3 family members. Taken together, these cuproenzyme analyses indicate an overall functional mismatch between protein levels and copper-dependent activity concomitant with changes in copper distribution and copper handling gene expression in the MS spinal cord.

**Fig. 4:**
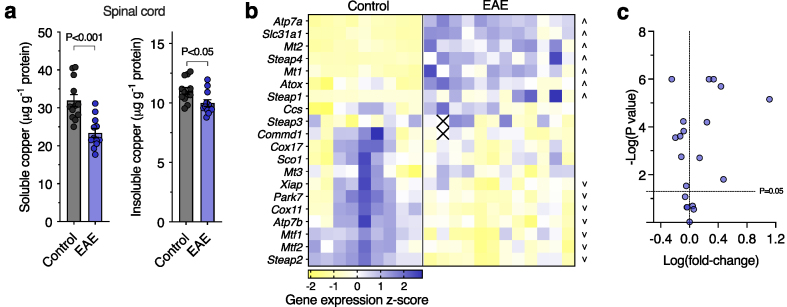
**Copper and copper handling gene expression in the experimental autoimmune encephalomyelitis (EAE) mouse model**. (a) Quantitation of copper partitioning into TBS-soluble and -insoluble fractions from EAE spinal cord samples. (b) Z-score heatmap showing expression of genes encoding copper transporters and chaperones in control and EAE mouse spinal cord measured by quantitative RT-PCR. Squares in transcript heatmaps represent z-scores for individual control and EAE mice. Up and down arrowheads identify genes with significantly increased or decreased expression in EAE mice, respectively. Crosses in heatmaps represent excluded values. (c) Volcano plot of data from (b) highlighting genes that are significantly up- or down-regulated using a P ​< ​0.05 threshold labelled as a dotted line. Circles in bar graphs represent individual mice. Bar graphs presented as mean values ​± ​SEM with labelled P values indicating statistically significant differences.

To investigate whether copper-related changes in the MS spinal cord may represent a potential therapeutic target we assessed the CNS-permeant bis(thiosemicarbazone)-copper complex Cu^II^(atsm) [33] in the experimental autoimmune encephalomyelitis (EAE) model. Alongside other proposed modes of action, Cu^II^(atsm) acts as a copper modulating compound which has been shown to be neuroprotective in diverse mouse models of neurodegeneration [[34], [35], [36], [37], [38], [39], [40], [41], [42]]. Moreover, Cu^II^(atsm) has recently been shown to be beneficial in the cuprizone mouse model of MS [20] lending support for copper perturbations in MS being a potential therapeutic target amenable to treatment with Cu^II^(atsm). The EAE model is utilised in MS research for its involvement of spinal cord pathology and physical manifestation of neurological symptoms. By exhibiting features of demyelination, neuronal damage and degeneration, in addition to permanent neurological disability [29,[43], [44], [45]], EAE mice represent a valuable tool for broadly modelling aspects of MS.

In line with the human MS spinal cord, levels of copper were found to be decreased in the TBS-soluble fraction of the EAE spinal cord, but in contrast with changes seen for the MS spinal cord, the EAE mice also showed a decrease in TBS-insoluble copper levels (Fig. 4a). These copper changes translated into gene expression differences with 14 of the 20 measured copper-handling genes displaying altered expression in the EAE spinal cord (Fig. 4b and c; Additional file 1: Fig. S6). Whilst the EAE copper-handling gene expression profile showed differences compared to human MS, these analyses demonstrate that copper perturbations are observed in both human cases and the model of disease.

**Fig. 5:**
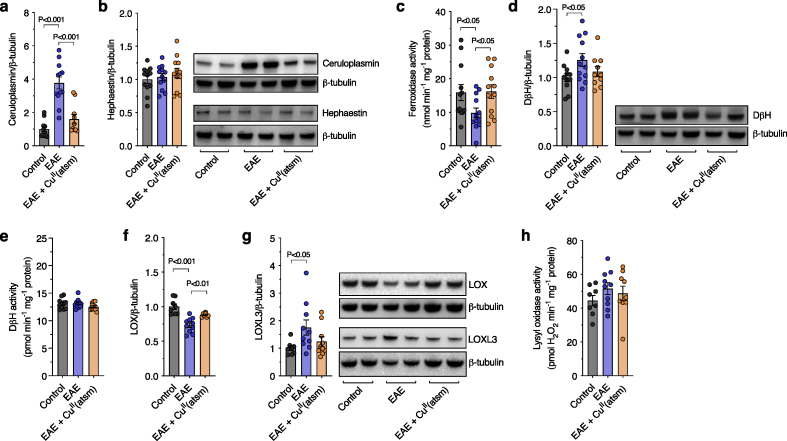
**Cuproenzyme functionality in the EAE mouse model**. (a, b) Protein levels of the ferroxidases ceruloplasmin and hephaestin in spinal cord samples from control and EAE mice, and EAE mice treated with Cu^II^(atsm), determined by western blot and expressed relative to control mice. (c) Spinal cord ferroxidase activity expressed as nmol holo-transferrin produced min^-^^1^ mg^-^^1^ sample protein. (d) DβH protein levels determined by western blot and expressed relative to control mice. (e) Activity of DβH expressed as pmol norepinephrine produced min^-^^1^ mg^-^^1^ sample protein relative to control mice. (f, g) Protein levels of LOX and LOXL3 determined by western blot and expressed relative to control mice. (h) Total lysyl oxidase activity expressed as nmol H_2_O_2_ produced min^-^^1^ mg^-^^1^ sample protein relative to control mice. Circles in bar graphs represent individual mice. Bar graphs presented as mean values ​± ​SEM with labelled P values indicating statistically significant differences.

Consistent with the cuproenzyme changes observed in human MS, ceruloplasmin levels were elevated despite a decrease in total ferroxidase activity, while hephaestin levels were unchanged (Fig. 5a–c). Similarly, DβH protein levels were elevated without a commensurate increase in activity (Fig. 5d and e) while LOX and LOXL3 levels were decreased and increased, respectively, translating into unchanged levels of overall LOX activity (Fig. 5f–h). To assess whether this functional cuproenzyme output could be restored pharmacologically, EAE mice were also treated with Cu^II^(atsm) resulting in a normalisation of protein and activity levels (Fig. 5a–h) alongside augmented copper levels (Additional file 1: Fig. S7a). Together, these results show broad commonalities across model and disease in terms of functional copper-dependent changes associated with spinal cord copper perturbations. Moreover, initial support is provided for being able to target these functional outputs pharmacologically in an MS context using the copper-modulating compound Cu^II^(atsm).

**Fig. 6:**
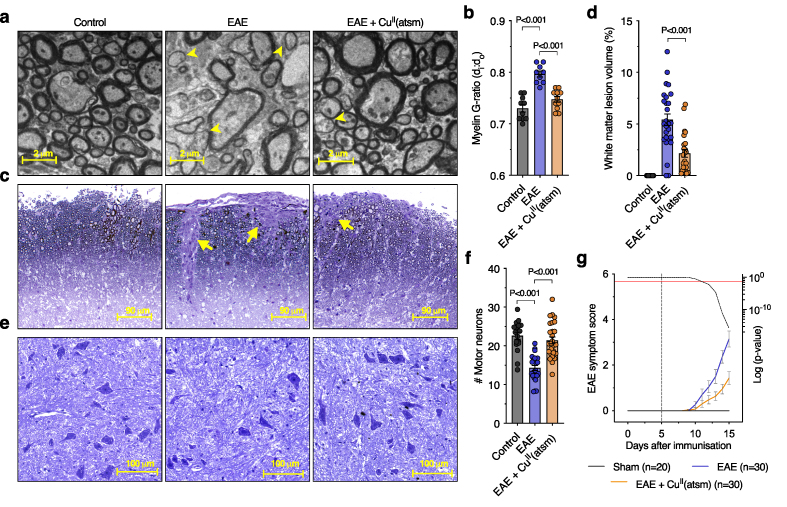
**Effects of Cu^II^(atsm) on myelin integrity and motor neuron numbers in EAE mice**. (a) Representative electron microscopy images for myelination of axons in the spinal cord of control and EAE mice, and EAE mice treated with Cu^II^(atsm). Yellow arrowheads highlight regions of demyelination. (b) Myelination of axons in the spinal cord expressed as G-ratio with higher values corresponding to demyelination. (c) Representative toluidine blue microscopy images showing white matter lesions (yellow arrows) in the spinal cord of control and EAE mice, and EAE mice treated with Cu^II^(atsm). (d) Quantitation of white matter lesion volumes expressed % of total white matter volume. (e) Representative toluidine blue microscopy images showing intensely stained motor neuron soma in the ventral grey matter of control and EAE mice, and EAE mice treated with Cu^II^(atsm). (f) Quantitation of motor neuron numbers. (g) Change in EAE symptom score up until the point of tissue collection for control and EAE mice, and EAE mice treated with Cu^II^(atsm). Vertical dotted line indicates initiation of Cu^II^(atsm) treatment with statistical significance highlighted by the dotted black line crossing below the horizontal red line. Circles in bar graphs represent individual mice. Bar graphs presented as mean values ​± ​SEM with labelled P values indicating statistically significant differences.

In parallel with biochemical changes related to cuproenzyme function, the EAE spinal cord showed clear manifestation of axonal demyelination evident in G ratio changes (Fig. 6a and b; Additional file 1: Figs. S7b–f), white matter lesion volumes (Fig. 6c and d), and Luxol fast blue staining (Additional file 1: Fig. S7g) in addition to a decrease in levels of the myelin protein CNPase comparable to human MS (Additional file 1: Figs. S7h and i). Administering Cu^II^(atsm) to the EAE mice resulted in improvement of features of demyelination and lesion volume (Fig. 6a–d; Additional file 1: Figs. S7b–i). Assessment of spinal cord ventral grey matter revealed a 36% decrease in the number of motor neurons in the EAE mice (Fig. 6e and f) as per previous findings [29], indicating the pertinence of this model to neurodegeneration in MS. Of the remaining motor neurons in the EAE mice soma area was unchanged whilst the diameter of axons projecting into the white matter was decreased (Additional file 1: Fig. S8). Treatment with Cu^II^(atsm) abrogated motor neuron loss in EAE spinal cord (Fig. 6e and f) and displayed an attenuation of EAE symptom score (Fig. 6g) up until tissue collection for all analyses. Given that administration of Cu^II^(atsm) commenced in the EAE mice on day 5 (i.e., after immunisation but before development of physical signs of disease) this raised the possibility that any observed Cu^II^(atsm)-mediated effects could be due to suppression of disease induction via peripheral lymphoid organs, thereby providing indirect beneficial effects for the CNS. To assess this possibility, we examined expression of genes related to immune responses in spleen samples. Consistent with MOG_35-55_ peptide immunisation inducing an immune response in the spleen, expression of *Il1a*, *Nos2*, *Tmem119*, *Il1b*, and *Mt1* were all elevated in EAE mouse spleen relative to control mouse spleen (Additional file 1: Fig. S9). The absence of any effect of Cu^II^(atsm) on these gene expression changes in the spleen indicates the compound is not mediating its protective effects through indirect actions in peripheral lymphoid organs. These findings provide initial support for the neuroprotective capacity of Cu^II^(atsm) in an MS context and a basis for further examination of Cu^II^(atsm) as a potential therapeutic option in MS.

## Discussion

Perturbations to copper and copper-related processes have been broadly implicated in pathological features and symptoms related to MS. Insufficient copper availability in humans is documented in the form of copper deficiency myelopathy, a disorder which manifests as neurological symptoms associated with demyelination, white matter lesions, and axonopathy in the CNS [46,47]. Without treatment, patients with copper deficiency myelopathy suffer from a progressive worsening of symptoms and pathology with similarities to MS [48]. These MS-like symptoms and pathological features are remedied in copper deficiency myelopathy through copper supplementation and modification of risk factors which primarily relate to impairment of copper absorption in the upper gastrointestinal tract. Congruent with this, hypomyelination in copper-deficient rats is remedied by dietary copper replacement [49]. More recently, histological assessment of SLC31A1, ATP7A, and ATP7B in post-mortem human tissue has indicated that copper-related abnormalities are present in MS-affected white matter, with lesions appearing more affected than normal appearing white matter [16]. The relevance of copper to MS pathology is further illustrated by administration of the copper chelator cuprizone to rodents to model key features of the disease, including demyelination in the CNS [[50], [51], [52], [53], [54], [55]]. Although some reports suggest formation of a toxic cuprizone-copper complex as driving demyelination in the cuprizone model rather than copper depletion *per se* [56], studies that measured copper report decreased copper levels in affected brain regions of cuprizone treated animals [20,57]. But despite these lines of evidence indicating a role for copper in MS and MS-related pathologies, the relationship between copper and MS remains insufficiently examined and understood. Not least of which, reports describing direct assessment of copper in human, MS-affected CNS tissue do not exist, and the therapeutic potential of targeting copper perturbations in MS has been largely unexplored. In this study, we show that human MS cases display broad changes to copper-related factors spanning levels and anatomical distribution, gene expression, and downstream functional consequences. We also provide evidence that features of these copper perturbations are recapitulated in the EAE model and may represent potential targets of interest for human MS.

The fundamental requirement for copper in neurological function is exemplified by Menkes disease where mutations affecting the copper transporter ATP7A result in systemic copper deficiency, cuproenzyme dysfunction, neurodegeneration, and premature death [58]. Mutations affecting ATP7A also cause occipital horn syndrome [59] and a rare form of adult-onset motor neuropathy [60], further emphasising the need for available copper in the CNS. A heightened sensitivity of the CNS to copper perturbations is consistent with its naturally slow turnover rate within the CNS compared to peripheral tissues [61]. This is particularly evident in the context of the neurodegenerative disease amyotrophic lateral sclerosis (ALS) where ubiquitous over-expression of mutant SOD1 in transgenic mice creates an elevated requirement for copper, and while peripheral tissues are able to meet this demand, the CNS does not [62]. Consequently, mutant SOD1 accumulates in a copper-deficient state in these mice over time selectively in the CNS [62,63]. Conversely, excessive copper levels in the CNS are also pernicious as demonstrated in Wilson's disease in which mutations to the copper transporter ATP7B result in abnormal CNS copper deposition and neurodegeneration [64]. Ultimately, the CNS relies upon a fine balance of copper levels where pathological alterations have the capacity to drive a wide range of neurological problems.

In the context of MS, excess copper has been implicated in white matter demyelination and oligodendrocyte loss due to an upregulation of copper transporters CTR1, ATP7A, and ATP7B by activated astrocytes prompting copper uptake and release [16]. These localised changes to disease-affected white matter regions were observed in both human MS cases and mouse models of MS, including EAE and cuprizone-treated mice. Here, we show that while bulk copper is elevated in the MS spinal cord, further analyses reveal nuanced shifts in copper distribution. In particular, while the white matter indicated an increase in copper levels in support of previously reported changes to copper transporter expression [16], the ventral grey matter displayed a marked decrease. These observations harbour similarities to those recently reported for the ALS-affected spinal cord which also showed an elevation in white matter copper coupled with a decrease in the ventral grey matter [65]. Why the white and grey matter are differentially affected remains to be established. Nonetheless, these results indicate a complexity of copper metallobiology beyond mere excess and deficiency in MS and broader neurodegeneration. This is congruent with our observation of decreased copper levels in TBS-soluble extracts of spinal cord tissue yet elevated levels of copper in the TBS-insoluble fraction. The TBS-soluble fraction is enriched for cytoplasmic proteins (Additional file 1: Fig. S1), and as copper trafficking proteins responsible for metallation of cuproenzymes reside in the cytoplasm, diminished levels of copper in this fraction could support a decreased amount of bioavailable copper which is reflected in the perturbed activities of cuproenzymes in MS. Conversely, it is conceivable that in MS where lesions are formed that copper becomes associated with insoluble components of these lesions. Analogous sequestration of copper into biologically inaccessible pools is described for the Alzheimer's disease-affected brain where copper accumulates in extracellular amyloid plaques [66]. Sequestration into a biologically inaccessible pool having an impact on copper-dependent processes within the CNS is in line with the natural turnover of copper being particularly slow in the CNS when compared to other tissues [61]. In other words, accumulation of copper within the white matter and/or TBS-insoluble fraction in MS would directly affect copper-dependent processes in other regions or fractions because the CNS has limited capacity to respond to altered copper requirements. The factors that prompt biochemical and spatial redistribution of copper in MS require further exploration.

In parallel with observed copper changes, the MS spinal cord also displayed broad perturbations to copper-related gene expression including previously reported alterations in levels for copper transporters CTR1, ATP7A, and ATP7B. Curiously, the gene expression profiles for the white and grey matter showed a considerable amount of alignment despite deviations in copper distribution and may represent a general response to alterations in copper levels. Copper gene expression was also perturbed in the EAE spinal cord, further indicating a copper problem in this model alongside human MS. However, distinctions were observed between human cases and the EAE model where insoluble copper levels were elevated in the human tissue but decreased in the mice. This discrepancy, as well as differences in gene expression profile, could be a reflection of the model or the end-stage nature of the human tissue which was not manifest in the EAE model. In particular, the EAE mice used in this study involved a short timeframe for assessment in order to identify copper perturbations during acute stage of pathology and symptom progression. Comprehensive assessment of the EAE model throughout evolution of MS-like signs of disease is needed, including assessment at late stages of the model that will be more likely to align with outcomes from post-mortem human tissue. Further studies could also help clarify the respective contributions of white matter lesions and neurodegeneration to the neurological deficits that manifest in the EAE model, potentially involving, for example, targeted manipulation of an important copper transporter such as ATP7A prior to induction of the immune response.

Following from alterations to copper and copper-related gene expression, cuproenzyme functionality was found to be affected through a mismatch in activity and protein levels for ferroxidases ceruloplasmin and hephaestin, members of the lysyl oxidase family LOX and LOXL3, and DβH. Each of these cuproenzymes were chosen based on their roles in iron trafficking, extracellular matrix remodelling, and neurotransmitter metabolism, respectively, which have been implicated in MS pathology [[67], [68], [69]]. These cuproenzyme changes were also observed in the EAE spinal cord, supporting analogous disruptions to functional output as a result of copper perturbations. Of note, this mismatch between cuproenzyme levels and activity associated with copper changes has previously been reported in the CNS of both ALS [63,65] and Parkinson's disease [70,71] and may represent a common thread in neurodegeneration amenable to therapeutic intervention.

The copper modulating compound Cu^II^(atsm) has undergone extensive preclinical investigation in numerous mouse models of neurodegenerative disease [[34], [35], [36], [37], [38], [39], [40], [41], [42]], as well as the cuprizone model of MS [20], having shown therapeutic benefit as a neuroprotective agent. Amongst other mechanisms of action such as peroxynitrite detoxification [34,35] and targeting ferroptosis [72,73], Cu^II^(atsm) has been largely investigated as a copper modulator with capacity to restore cuproenzyme function in CNS tissues affected by copper perturbations [[37], [38], [39]]. As a result of promising preclinical findings Cu^II^(atsm) is currently under investigation at therapeutic doses for the treatment of ALS and Parkinson's disease (NCT02870634; NCT4082832; NCT03204929).

Results for MS cases and the EAE model being comparable to those observed in human ALS [65] and ALS mouse models [[37], [38], [39],^63^] prompted us to investigate whether copper perturbations in this context could be a pharmacological target for Cu^II^(atsm). Herein, our preliminary assessment of Cu^II^(atsm) in EAE mice showed a restoration of cuproenzyme functionality compared to sham-treated mice. Administration of Cu^II^(atsm) was also protective against spinal cord demyelination as well as motor neuron loss in EAE mice, highlighting its potential utility as a neuroprotective agent in MS. Whilst these outcomes show early promise, the treatment paradigm implemented in this study means that peripheral activity of Cu^II^(atsm), such as mitigation of disease induction occurring in peripheral lymphoid organs, could not be excluded as a possible explanation for observed improvements. To this end, we provide results that indicate the protective activity of Cu^II^(atsm) was not due to peripheral suppression of the autoimmune response necessary for induction of the MS-like phenotype (Additional file 1: Fig. S9). Nonetheless, treating EAE mice after the onset of signs of disease will further expand our understanding of the potential therapeutic utility of this compound in the context of MS, as established for ALS where the compound is neuroprotective within the CNS in mouse models of ALS when administered after the onset of signs of neurodegeneration [39,73]. Thus, subsequent investigation of Cu^II^(atsm) efficacy in the EAE model would need to entail post-symptomatic initiation of treatment and assessment throughout different phases of disease. Moreover, alternative model systems could also be considered such as the Biozzi or NOD mice [74,75] that seek to reproduce chronic progressive aspects of MS for which neuroprotection remains a significant unmet need.

A salient consideration for the use of copper modulating compounds as a therapeutic strategy that merits caution is the prospect of toxicity arising from excess copper levels. In MS, this concept has been investigated showing that white matter pathology involves increased expression of copper transporters associated with activated astrocytes and that copper released by astrocytes is harmful to oligodendrocytes [16]. Additionally, excess copper has been found to drive aggregation of lipoylated mitochondrial proteins through direct interaction with the lipoyl moiety with toxic repercussions [76]. Taking these factors into account, ensuring prospective copper modulating therapies avoid unintended toxic consequences resulting from excess will be of paramount importance. An advantage of Cu^II^(atsm) lies in its chemistry where its copper modulating properties are dictated by conditions related to a perturbed intracellular redox environment [77]. In the clinic, isotopically labelled Cu^II^(atsm) has been used to image affected CNS regions of patients with neurodegenerative disease including ALS and Parkinson's disease [[78], [79], [80], [81]]. Selective retention of the Cu^II^(atsm)-delivered, PET-detectable copper signal in affected regions of the CNS is attributed to alterations to the cellular redox environment associated with pathology in these diseases [[77], [78], [79], [80], [81], [82], [83]]. Although copper associated with Cu^II^(atsm) is detectable in peripheral tissue [39,84], preferential retention and copper modulating actions where it is required is supported by the ability to restore cuproenzyme functionality in disease-affected regions [[37], [38], [39]]. These properties are likely to provide benefit in contrast to other copper related agents that are characterised by a higher propensity towards copper release under physiological conditions [77].

The involvement of neurodegeneration in the worsening of permanent neurological disability in MS underscores the necessity for neuroprotection in therapeutic regimens. Progress on this front, however, has been limited to date with new approaches required through an expanded understanding of potential therapeutic targets amenable to pharmacological intervention. Here, we describe copper perturbations as a broad disease feature of MS and provide initial preclinical outcomes in the EAE model supporting a neuroprotective role for Cu^II^(atsm). These findings highlight a role for altered copper biology in MS and suggest that further investigation of Cu^II^(atsm) is warranted as a potential therapeutic treatment for neurodegeneration in MS.

## Ethics approvals

All research involving mice was approved by a University of Melbourne Animal Experimentation Committee (approval numbers 1513554 and 1613995) and conformed with guidelines of the Australian National Health and Medical Research Council. All procedures involving the use of post-mortem human tissue were approved by a University of Melbourne Human Research Ethics Committee (Project ID 1238124) and adhered to guidelines of the Australian National Health and Medical Research Council.

## Consent for publication

Human tissues were donated to the respective tissue banks with informed consent.

## Availability of data and materials

All data supporting the conclusions of this article are included within the article and in the additional file provided.

## Funding

The research was supported by funds from 10.13039/501100000924Multiple Sclerosis Research Australia, The 10.13039/501100019999Trish Multiple Sclerosis Research Foundation, The National 10.13039/100018696Health and 10.13039/501100000265Medical Research Council, The Michael Hirshorn Medical Research Commercialisation Fund, and 10.13039/501100021779Perpetual Trustees (Margaret Dawn Marks Trust). JBWH was a recipient of an Australian Postgraduate Award and the Nancy Frances Curry Scholarship. JRL was a recipient of an NHMRC Early Career Fellowship. PSD was a recipient of an Australian Research Council Future Fellowship (FT3). DJH was a recipient of an NHMRC Industry Career Development Fellowship (CDF1) in partnership with Agilent Technologies. ARW was a recipient of an NHMRC Senior Research Fellowship. PJC was a recipient of an NHMRC Career Development Fellowship (CDF2, 1084927).

## Author Contributions

JBWH, KK and JRL designed experiments, performed experiments, and analysed data. SWM, CR, DJH and BP performed experiments and analysed data. CAM collected and performed neuropathological assessment of human tissue samples. GB and PSD synthesised Cu^II^(atsm). TJK, SSM, JSB, SA, AIB, PSD, ARW and BRR interpreted results and provided important experimental guidance. PJC conceived the project, coordinated the study, designed experiments, performed experiments, and analysed data. JBWH and PJC wrote the draft of the manuscript; all authors edited and approved the final version.

## Declaration of competing interest

Collaborative Medicinal Development has licensed intellectual property pertaining to Cu^II^(atsm) from the University of Melbourne where the inventors include ARW and PSD. AIB is a paid consultant for Collaborative Medicinal Development LLC and has a profit share interest in Collaborative Medicinal Development Pty Ltd. PJC and JSB are unpaid consultants for Collaborative Medicinal Development LLC. DJH received research and material support from Agilent Technologies and ESI Ltd.
