# Supplementary material for: Integrated elemental analysis supports targeting copper perturbations as a therapeutic strategy in multiple sclerosis
Source: Neurotherapeutics. 2024 Aug 19;21(5):e00432. doi: 10.1016/j.neurot.2024.e00432 (PMC11579877; doi:10.1016/j.neurot.2024.e00432)
Supplement: Multimedia component 1 [file mmc1.pdf]

Additional file 1:

Table S1. Demographics of control and MS cases.

Sex	Control	Total MS cases	Primary progressive MS	Secondary progressive MS
Male	7 (64%)	14 (52%)	3 (50%)	11 (52%)
Mean age ± S.D.	74 ± 13.8	48.5 ± 9.9	43.5 ± 5.8	49.8 ± 10.6
Female	4 (36%)	13 (48%)	3 (50%)	10 (48%)
Mean age ± S.D.	88.9 ± 0.2	57.6 ± 11.9	51.8 ± 15.1	59.3 ± 11.1

Table S2. Mass spectrometry parameters used in detection and quantitation of DβH activity.

Compound	Precursor ion $[M+H]^+$	Product ion m/z	Declustering potential (V)	Collision energy (eV)
Dopamine	154.1	137.1	29	10
Norepinephrine	170.1	152.1	20	10
Epinephrine	184.1	166.1	15	8

Table S3. Copper handling genes analysed in human and mouse spinal cord samples with NCBI gene identification number and Taqman assays used for qPCR analyses.

Gene	Species	NCBI gene ID[1]	Taqman Assay ID	Gene	Species	NCBI gene ID[1]	Taqman Assay ID
ATOX1	Human	475	Hs00187841_m1	*Atox1*	Mouse	11927	Mm00839626_m1
ATP7A	Human	538	Hs00163707_m1	*Atp7a*	Mouse	11977	Mm00437663_m1
ATP7B	Human	540	Hs01075310_m1	*Atp7b*	Mouse	11979	Mm00599675_m1
CCS	Human	9973	Hs00192851_m1	*Ccs*	Mouse	12460	Mm00444148_m1
COMMD1	Human	150684	Hs00415059_m1	*Commd1*	Mouse	17486	Mm01239669_m1
COX11	Human	1353	Hs00362087_m1	*Cox11*	Mouse	69802	Mm01615963_g1
COX17	Human	10063	Hs01053235_g1	*Cox17*	Mouse	12856	Mm01346225_m1
MT1A	Human	4489	Hs00831826_s1	*Mt1*	Mouse	17748	Mm00496660_g1
MT1G	Human	4495	Hs04401199_s1	*Mt2*	Mouse	17750	Mm00809556_s1
MT2A	Human	4502	Hs02379661_g1	*Mt3*	Mouse	17751	Mm00496661_g1
MT3	Human	4504	Hs00359394_g1	*Mtf1*	Mouse	17764	Mm00485274_m1
MTF1	Human	4520	Hs00232306_m1	*Mtf2*	Mouse	17765	Mm00489151_m1
MTF2	Human	22823	Hs00980938_m1	*Park7*	Mouse	57320	Mm00498538_m1
SCO1	Human	6341	Hs01552201_m1	*Sco1*	Mouse	52892	Mm01329074_m1
SLC31A1	Human	1317	Hs00977266_g1	*Slc31a1*	Mouse	20529	Mm00558247_m1
STEAP1	Human	26872	Hs00185180_m1	*Steap1*	Mouse	70358	Mm00459097_m1
STEAP2	Human	261729	Hs00401292_m1	*Steap2*	Mouse	74051	Mm01320129_m1
STEAP3	Human	55240	Hs00217292_m1	*Steap3*	Mouse	68428	Mm01287243_m1
STEAP4	Human	79689	Hs01026584_m1	*Steap4*	Mouse	117167	Mm00475405_m1
XIAP	Human	331	Hs00745222_s1	*Xiap*	Mouse	11798	Mm00776505_m1

[1]NCBI gene identification numbers from https://www.ncbi.nlm.nih.gov/gene/

Table S4. Immune response genes analysed in mouse spleen samples with NCBI gene identification number and Taqman assays used for qPCR analyses.

Gene	Species	NCBI gene ID[1]	Taqman Assay ID
Il1a	Mouse	16175	Mm00439620_m1
Nos2	Mouse	18126	Mm00440502_m1
Tmem119	Mouse	231633	Mm00525305_m1
Il1b	Mouse	16176	Mm00434228_m1
Mt1	Mouse	17748	Mm00496660_g1

[1]NCBI gene identification numbers from https://www.ncbi.nlm.nih.gov/gene/

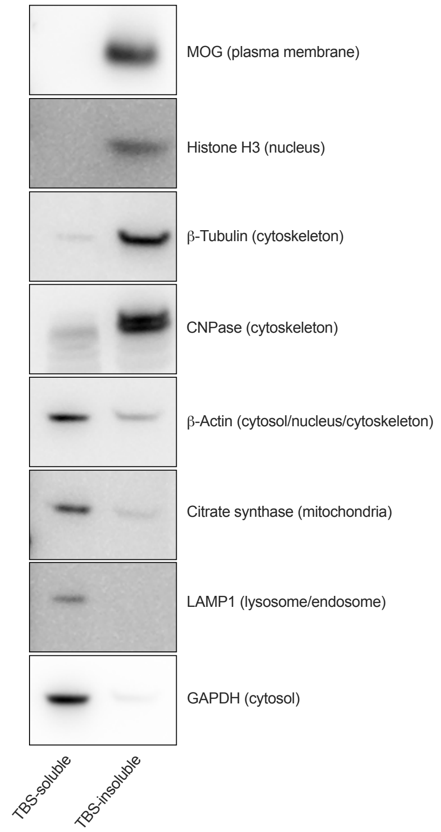

Fig. S1. Partitioning of endogenous proteins in human spinal cord into TBS-soluble and TBS-insoluble fractions. Proteins that partition into the TBS-soluble fraction are primarily cytoplasmic excepting the cytoskeleton. Proteins that partition into the TBS-insoluble fraction are primarily associated with the plasma membrane, nucleus, or cytoskeleton. Some cross over is evident for proteins such as β-actin that are more dispersed throughout the cell.

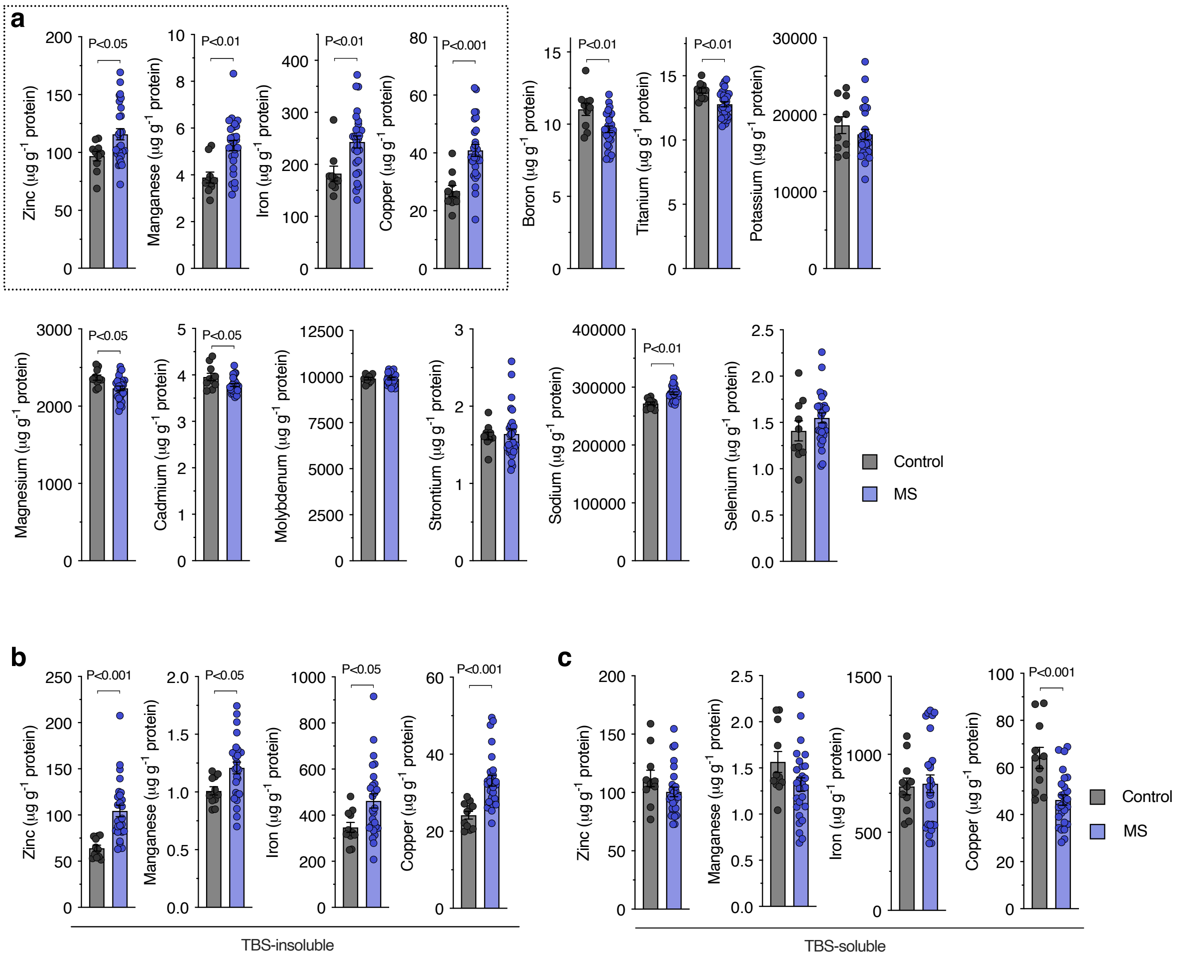

Fig. S2. Elemental composition of the human MS-affected spinal cord. (**a**) Total (bulk) tissue content of endogenous elements in spinal cord samples expressed relative to protein content. Highlighted elements (zinc, manganese, iron and copper) are those which displayed a ≥ 1.2-fold change in MS cases to controls. (**b,c**) Fraction partitioning of zinc, manganese, iron, and copper in TBS-soluble and -insoluble fractions for MS cases and controls expressed relative to protein content. Circles in bar graphs represent individual MS and control cases. Bar graphs presented as mean values ± SEM with labelled P values indicating statistically significant differences.

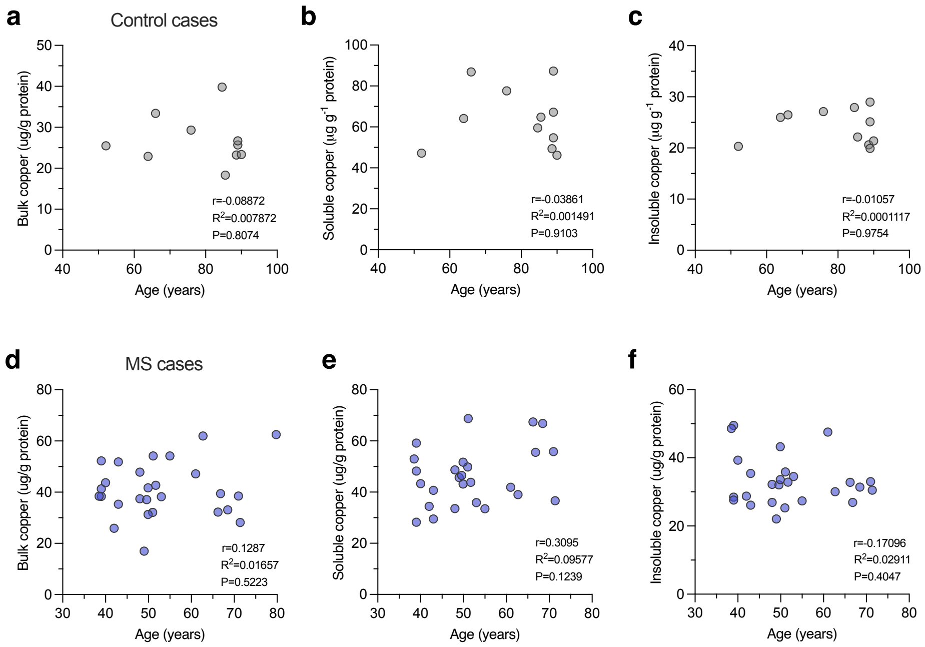

Fig. S3. Relationship between spinal cord copper levels and age. (**a-f**) Spinal cord copper measured as total, TBS-soluble, and TBS-insoluble levels show no relationship with age in control (**a-c**) and MS cases (**d-f**). Strength of relationships was assessed using the Pearson correlation coefficient (r), R^2, and statistical significance.

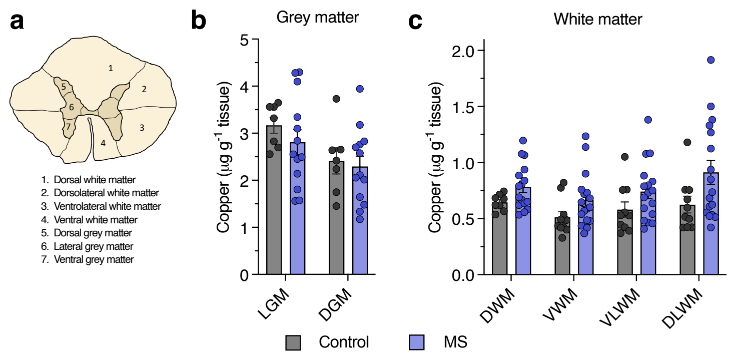

Fig. S4. Anatomical distribution of copper in the human MS-affected spinal cord. (**a**) Illustration of spinal cord anatomical regions of interest used for quantitation. (**b**) Copper in grey matter (LGM: lateral grey matter; DGM: dorsal grey matter) regions of interest. (**c**) Copper in white matter (DWM: dorsal white matter, VWM = ventral white matter; VLWM = ventrolateral white matter; DLWM = dorsolateral white matter) regions of interest. Circles in bar graphs represent individual MS and control cases. Bar graphs presented as mean values ± SEM with labelled P values indicating statistically significant differences.

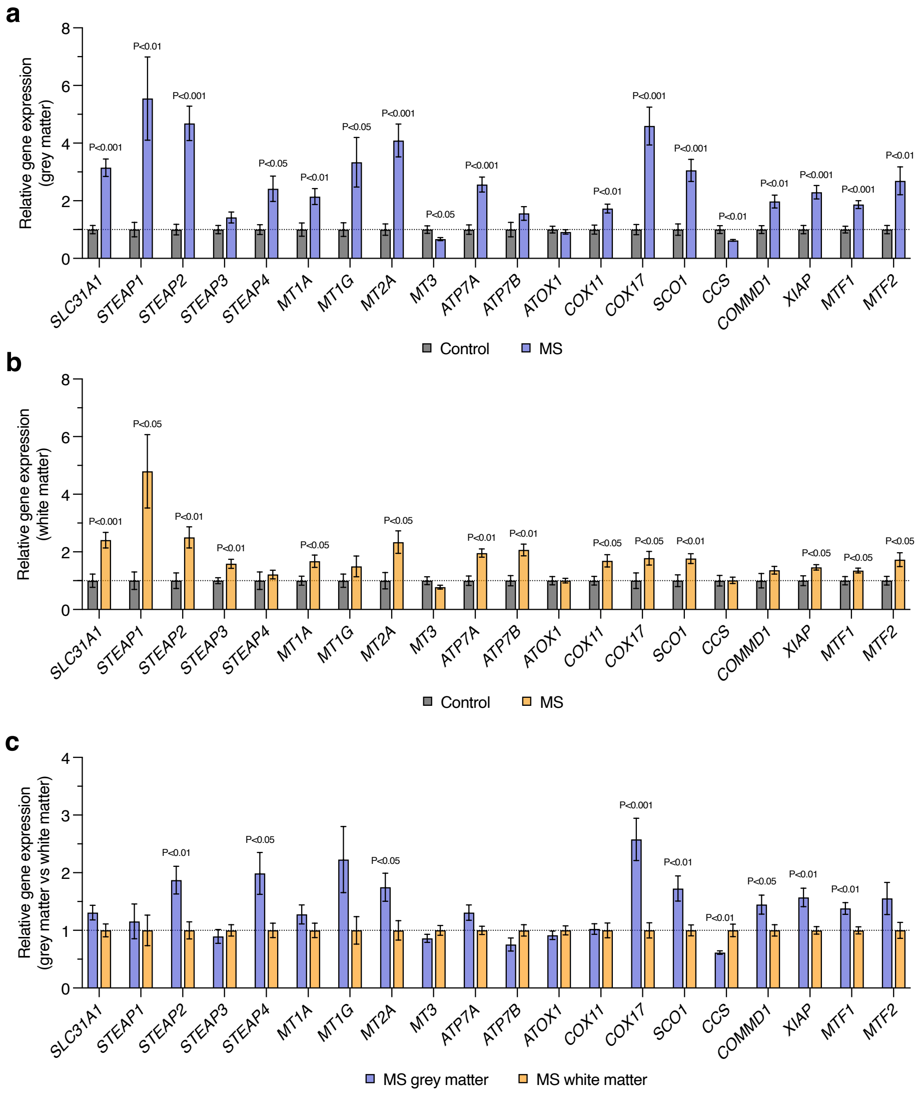

Fig. S5. Expression of copper handling genes in the human MS-affected spinal cord. (**a**,**b**) Expression of individual genes in grey matter (**a**) and white matter (**b**) extracts analysed via quantitative RT-PCR and expressed relative to controls. (**c**) Expression of individual genes in MS cases presented as expression in the grey matter relative to the white matter. Bar graphs presented as mean values ± SEM with labelled P values indicating statistically significant differences.

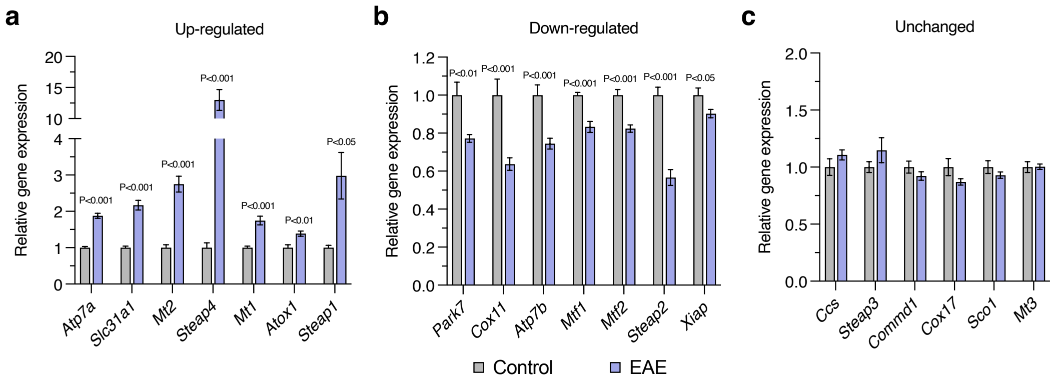

Fig. S6. Expression of copper handling genes in the EAE mouse spinal cord. (**a-c**) Expression of individual genes analysed via quantitative RT-PCR and expressed relative to control mice. Genes significantly up-regulated (**a**), down-regulated (**b**), or unchanged (**c**) shown separately. Bar graphs presented as mean values ± SEM with labelled P values indicating statistically significant differences.

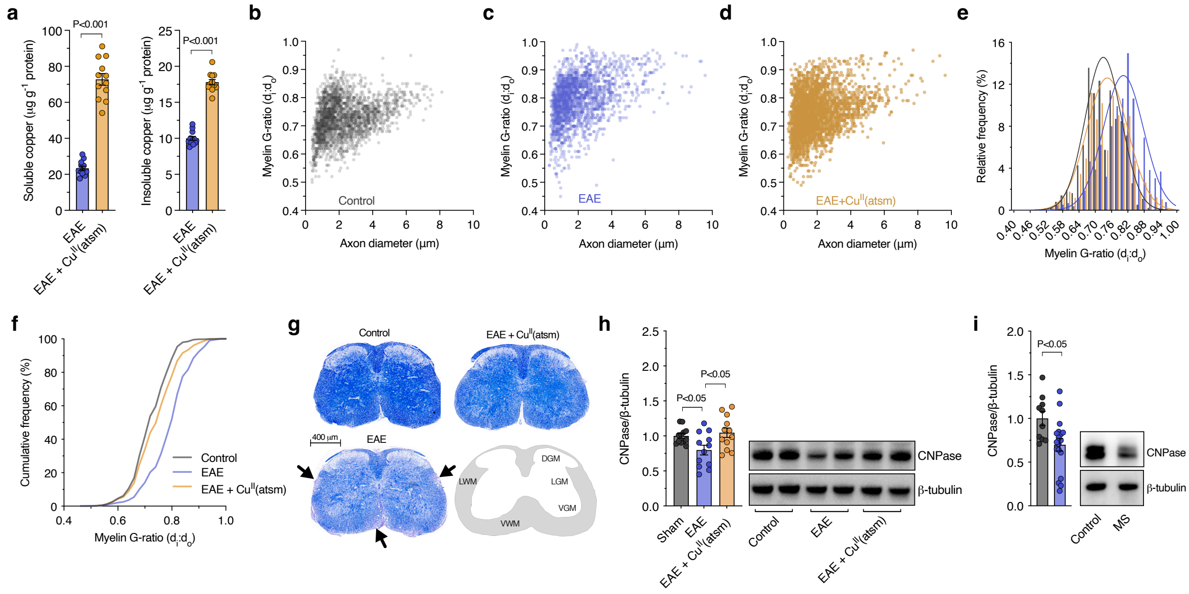

Fig. S7. Effects of Cu^{II}(atsm) on myelination in the EAE mouse spinal cord. (**a**) Copper levels in TBS-soluble and -insoluble spinal cord fractions for EAE sham-treated and Cu^{II}(atsm)-treated EAE mice. (**b-d**) Scatterplots of myelin G-ratio relative to axonal diameter in the spinal cord of control and EAE mice, and EAE mice treated with Cu^{II}(atsm). Dots represent individual axon measurements performed (n=2,260, 2,040 and 2,715 for **b**, **c** and **d**, respectively). (**e,f**) Myelin G-ratio data for control and EAE mice, and EAE mice treated with Cu^{II}(atsm), shown as relative frequency and cumulative frequency expressed as %. Fitted curves show Gaussian least squares fit. (**g**) Representative microscopy images for spinal cord myelination using Luxol fast blue staining in control and EAE mice, and EAE mice treated with Cu^{II}(atsm). Illustration of mouse spinal cord transverse sections with labelled regions (DGM: dorsal grey matter; LGM: lateral grey matter; VGM: ventral grey matter; LWM: lateral white matter; VWM: ventral white matter). (**h**) Spinal cord levels of the myelin protein CNPase determined by western blot in EAE sham-treated and Cu^{II}(atsm)-treated EAE mice expressed relative to control mice. (**i**) Spinal cord levels of CNPase determined by western blot in human MS cases expressed relative to controls. Circles in bar graphs represent mice or human cases. Bar graphs presented as mean values ± SEM with labelled P values indicating statistically significant differences.

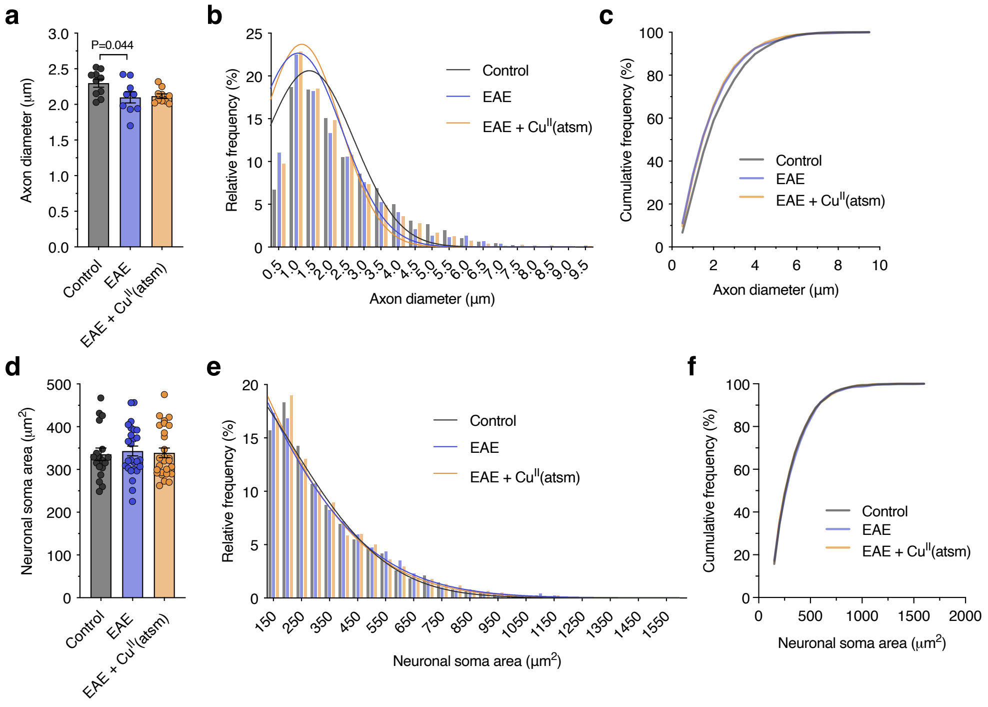

Fig. S8. Effects of Cu^{II}(atsm) on neuronal parameters in the EAE mouse spinal cord. (**a**) Quantitation of average axonal diameter in the spinal cord of control and EAE mice, and EAE mice treated with Cu^{II}(atsm). (**b,c**) Average axonal diameter in the spinal cord of control and EAE mice, and EAE mice treated with Cu^{II}(atsm) expressed as relative frequency and cumulative frequency. (**d**) Quantitation of average motor neuronal soma area in the ventral grey matter of control and EAE mice, and EAE mice treated with Cu^{II}(atsm). Average motor neuronal soma area in the ventral grey matter of control and EAE mice, and EAE mice treated with Cu^{II}(atsm) expressed as relative frequency and cumulative frequency. Circles in bar graphs represent mice. Bar graphs presented as mean values ± SEM with labelled P values indicating statistically significant differences. Fitted curves in **b**, **c**, **e** and **f** show Gaussian least squares fit.

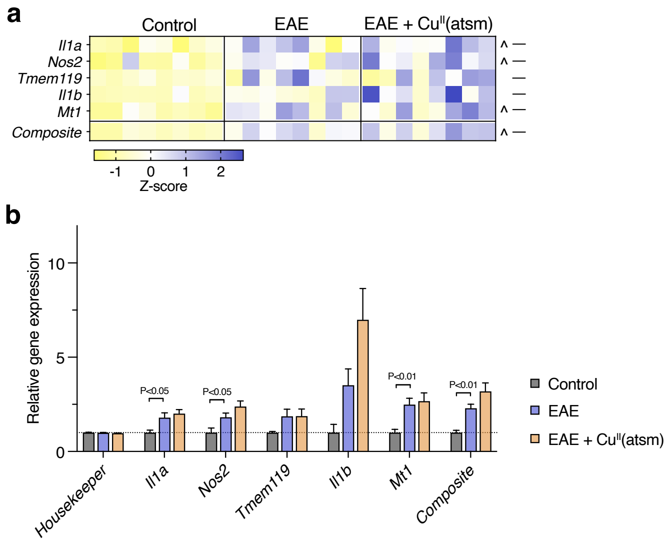

Fig. S9. Expression of immune response genes in mouse spleen. (**a**) Z-score heatmaps showing expression of immune response related genes in spleen samples measured by quantitative RT-PCR. Squares in transcript heatmaps represent z-scores for individual mice. Up arrowheads (^) identify genes with significantly increased expression in EAE mice compared to controls. Dashes (–) identify genes that are not changed in the Cu^{II}(atsm) treated EAE mice compared to EAE mice. (**b**) Expression of individual genes analysed via quantitative RT-PCR and expressed relative to control mice. Bar graphs presented as mean values ± SEM with labelled P values indicating statistically significant differences. 'Housekeeper' represents average of *Gapdh*, *Actb*, and *Tuba1a* which were used for all gene expression normalisation. Composite represents average of the target immune genes.
